# Health Risk and Quality Assessment of Vegetables Cultivated on Soils from a Heavily Polluted Old Mining Area

**DOI:** 10.3390/toxics11070583

**Published:** 2023-07-04

**Authors:** Daniela Pavlíková, Veronika Zemanová, Milan Pavlík

**Affiliations:** Department of Agroenvironmental Chemistry and Plant Nutrition, Faculty of Agrobiology, Food and Natural Resources, Czech University of Life Sciences Prague, Kamýcká 129, 165 00 Prague, Czech Republic; zemanovav@af.czu.cz (V.Z.); pavlikm@af.czu.cz (M.P.)

**Keywords:** cadmium, carcinogenic risk, *Daucus carota*, *Lactuca sativa*, lead, *Rapahanus sativus*, target hazard quotient, toxic elements, zinc

## Abstract

Three garden vegetables—radish, carrot and lettuce—were cultivated in a pot experiment using two soils from the Příbram area polluted mainly by cadmium (Cd), zinc (Zn), lead (Pb) and chromium (Cr). The soils of the Příbram district, Czech Republic, are heavily polluted as a result of the atmospheric deposition of toxic elements originating from historic lead–silver mining and smelting activities. The results showed that lettuce absorbed the highest amounts of toxic elements (Cd 28 and 30, Cr 12 and 13, Zn 92 and 205 mg·kg^−1^ DW), except Pb, which was higher in radish (30 and 49 mg·kg^−1^ DW). Changes in macronutrient contents in edible parts were not found, except for sulfur. A higher total free amino acids (fAAs) accumulation was shown in all vegetables in more contaminated soil, with the highest fAA content being in radish. A group of essential fAAs reached 7–24% of total fAAs in vegetables. The risk to human health was characterized using the target hazard quotient and total hazard index (HI). The cumulative effect of the consumption of vegetables with HI > 1 showed possible non-carcinogenic health effects for lettuce and carrot. HI decreased in the order Cd > Pb > Cr > Zn. The carcinogenic risk of toxic elements decreased in the order Cd > Cr > Pb (0.00054, 0.00026, 0.00003). These values showed a carcinogenic risk from the consumption of lettuce and carrot and confirmed that the adult population of the studied area is at high risk if lettuce and carrot cultivated in this area are consumed daily.

## 1. Introduction

A significant part of soil pollution caused by toxic elements originates from anthropogenic activities, mainly from mining and ore smelting. One of these heavily polluted areas in the Czech Republic is the Příbram region, about 60 km southwest of Prague, the capital of the Czech Republic. The main source of soil contamination has been reported as the atmospheric deposition of toxic elements originating from Ag-bearing galena (PbS) and sphalerite (ZnS) mining, followed by ore smelting and lead processing. The lead processing industry in this area has led to an increase in lead (Pb), arsenic (As), cadmium (Cd), chromium (Cr) and zinc (Zn) soil contamination due to the high content of these elements in parent rock [1,2]. These activities have been ongoing in the region since 1786. Local polymetallic ores have been exhausted, and the remaining mines were closed in 1978/1979 [3]. According to Šichorová et al. [1], the area of maximum soil contamination is up to 1.5 km from the chimney of the smelter.

Toxic elements are taken up by plants from the soil, and their contents in plant biomass are dependent on element concentration and soil conditions [4]. The uptake, translocation and accumulation mechanisms differ for various toxic elements, their chemical forms and the plant species. According to Fahr et al. [5], in most plants, 90% of the total Pb is accumulated in roots. Reddy et al. [6] also found a higher Pb content located in plant roots than in above-ground biomass. Bidar et al. [7] confirmed root vegetables as moderate Pb accumulators. Similarly, chromium (Cr) is mainly accumulated in plant roots and is immobilized in the vacuoles of root cells, which is a strategy of plants to cope with Cr toxicity [8]. Rapid growth and high transpiration rates allow leafy vegetables to accumulate more toxic elements than other vegetables. Among leafy vegetables, lettuce has the highest capacity of accumulating Cd from contaminated soil [7,9,10] and contributes the greatest to human health risk [11].

Toxic elements can reduce biomass yield and crop quality and disrupt the metabolism of cultivated plants. They can limit nutrient plant uptake, affect biomass growth by inhibiting chlorophyll synthesis and photosynthesis processes and induce an enzymatic antioxidant response to oxidative stress [10,12,13]. Toxic elements lead to an increase in reactive oxygen species (ROS), such as superoxide anions, hydrogen peroxide and hydroxyl radicals. The excessive production of ROS in plant tissue can damage major biomolecules, including membrane lipids, amino acids, proteins and nucleic acids [14,15]. Although Zn is an essential micronutrient required for many enzymes involved in numerous physiological and metabolic processes of plants, the excess uptake of this element causes phytotoxic effects similar to those of non-essential toxic elements, for example, nutrient imbalance, leaf chlorosis, photosynthesis impairment, lipid peroxidation, inhibition in ATP production during respiration, DNA damage due to excessive ROS generation and many more [16,17,18].

The regular consumption of vegetables is beneficial to the development, growth and health of humans. Vegetables are important components of the human diet as a source of essential elements, vitamins, fiber and substances with antioxidative effects. The bioaccumulation of toxic elements in edible parts of vegetables cultivated on contaminated soils results in their entry into the food chain and negatively affects consumers’ health [19]. The consumption of vegetables contaminated with these elements causes serious human health issues, such as gastrointestinal cancer, fragile immunological mechanisms, mental growth retardation and malnutrition [20]. Symptoms of chronic diseases provoked by toxic elements depend not only on element properties but also on its dose, as well as the duration of exposure.

The kidneys and the liver are the main organs in the human body that are especially sensitive to Cd toxicity. Cadmium can cause bone diseases by disturbing the metabolism of calcium, magnesium, Zn, copper and iron ions [10,21]. According to Salaskar et al. [22], vegetable foods contribute more than 70% of Cd intake in the human diet. Lead is a neurotoxic element. Chronic exposure to Pb can be associated with an increased risk of developing neurodegenerative diseases. Both elements are carcinogenic for humans. Chromium is an essential micronutrient for the human metabolism of glucose and lipids, but its high concentration (Cr^6+^) in the human body can also have a carcinogenetic effect [20]. The essential element Zn has a normal range for oral intake of 8–10 mg·day^−1^, and intake beyond this range is associated with either deficiency or toxicity. Zn excess is known to reduce immune function and high-density lipoprotein levels.

For this experiment, three vegetable species—radish, carrot and lettuce—were chosen, as they are commonly cultivated garden vegetables with a high consumption rate in the Czech Republic. The Příbram region is one of the most polluted areas by toxic elements in the Czech Republic. People living here suffer from a wide range of health problems. The main aim of our study was to evaluate the quality reduction in vegetables cultivated in a heavily polluted former mining area and estimate the potential health risks associated with human consumption of common vegetables grown in this region.

## 2. Materials and Methods

### 2.1. Pot Experiment and Plant Sample Analysis

Vegetable species—radish (*Rapahanus sativus* L. var. *sativus* Pers. ‘TERCIA’), carrot (*Daucus carota* L. ‘NANTES 5′) and lettuce (*Lactuca sativa* L. ‘ADINAL’)—were cultivated in a pot experiment using two soils from a heavily polluted old mining and smelting area (49°42′24″ N, 13°58′32″ E, Table 1, Figure S1). Each pot was filled with 5 kg of soil and was mixed with nutrients in doses of 0.5 g N, 0.16 g P and 0.4 g K per pot (applied as NH_4_NO_3_ and K_2_HPO_4_ solutions). A total of 4 replicates were used for each vegetable and soil (24 pots in total).

Seeds of carrot and radish were purchased from the SEMO a.s. company store (Czech Republic) and were sown directly into the soil in pots (15 seeds per pot). Thinning was performed after two true leaves developed, keeping ten seedlings in each pot. Pre-cultivated plants of lettuce were purchased from the garden center at the six true leaves stage and placed in the pots with contaminated soil at a rate of 1 plant per pot. Vegetables were grown under greenhouse conditions (natural light conditions; temperature 20–22 °C during the day and 15–18 °C at night; relative humidity, ~60%). The vegetables were harvested at different stages of maturity for each species: after 6 weeks of growth for lettuce, 15 weeks of growth for carrot and 7 weeks of growth for radish.

Vegetables were divided into roots and above-ground biomass for lettuce and carrot and into roots, tubers and above-ground biomass for radish. Parts of plants were weighed. Yields of vegetables cultivated on both soils were not significantly different, except for carrot. Therefore, the data were not discussed in this manuscript. They were added as Supplementary Figure S2. Each part was washed with distilled water, blotted dry with filter paper and weighed. Samples were partitioned for further analysis. One portion was immediately frozen in liquid nitrogen and stored at –80 °C until analysis of free amino acids (AAs), while the other portion was oven-dried (40 °C) to constant weight and homogenized for elemental analysis.

Homogenized material (0.5 ± 0.05 g of dry weight) was digested in 10 mL of a mixture of HNO_3_ and H_2_O_2_ (4:1, *v*/*v*) at 120–180 °C in an Ethos 1 device (MLS GmbH, Leutkirch im Allgau, Germany). After cooling, the digested sample was diluted to 50 mL with demineralized water. The contents of elements in the edible parts of vegetables were determined by an Agilent 720 inductively coupled plasma optical emission spectrometer (ICP-OES; Agilent Technologies Inc., Santa Clara, CA, USA) after low-pressure microwave digestion [24]. Certified reference material (CRM NIST 1573a Tomato leaves and CRM NIST 1570a Spinach leaves, Analytika^®^, Prague, Czech Republic) was mineralized under the same conditions for quality assurance (Table S1).

The content of free AAs in edible parts of vegetables was determined using a Hewlett Packard 6890N/5975 MSD gas chromatography–mass spectrometry system (GC-MS; Agilent Technologies Inc., Santa Clara, CA, USA) with a ZB-AAA 10 m × 0.25 mm AA analysis GC column after derivatization of extracts by an EZ:faast kit (Phenomenex, Torrance, CA, USA). Extraction was performed as previously described [25].

### 2.2. Evaluation of Bioaccumulation Factor and Risk Assessment

To evaluate the ability of vegetables to uptake toxic elements from contaminated soil into their edible parts, the bioaccumulation factor (BAF) was calculated by the following formula:

BAF = C_vge_/C_soil,_ (1)

and parameters used for calculation are explained in Table 2.

To evaluate health risk assessment, chronic daily intake of toxic elements (CDI) was calculated by the following formula:

CDI = (C_vge_ × IR ×EF × ED)/(ET × BW), (2)

Daily consumptions of individual vegetables (IR) used for calculation were published by the Ministry of Agriculture of the Czech Republic [26]: IR_lettuce_ 0.007 kg·person^−1^·day^−1^, IR_carrot_ 0.0225 kg·person^−1^·day^−1^ and IR_radish_ 0.001 kg·person^−1^·day^−1^. The parameters used for calculation are explained in Table 2.

As an estimation of the potential hazard to human health through the consumption of vegetables grown in toxic-element-contaminated soil, the target hazard quotient (THQ) was calculated by the following formula:

THQ = CDI/RfD,(3)

where the RfD for Cd, Pb, Zn and Cr are 0.001, 0.0035, 0.300 and 0.003 mg·kg^−1^·day^−1^ [27,28]. The HQ method was used to assess the noncarcinogenic risk. If the result is less than 1, there will not be any obvious risk.

The hazard index (HI) was calculated by the following formula:

HI = ΣHQi,(4)

The carcinogenic risk (CR) was calculated using the following formula:

CR = CDI × SF,(5)

where the SF for Cd, Pb and Cr are 0.38, 0.0085 and 0.50 mg·kg^−1^·day^−1^ [28]. The CR represents the potential carcinogenic risk through the consumption of carcinogenic elements (Cd, Pb and Cr).

Total carcinogenic risk (TCR) was calculated using the following formula:

TCR = ΣCRi,(6)

where TCR is the sum of the potential carcinogenic risk of each carcinogenic element.

### 2.3. Statistical Methods

Statistica 12.0 software (StatSoft, Tulsa, OK, USA) was used for the statistical processing of the results. The data of technical replicates (three per each analysis) were averaged for four independent biological repeats (pots) of each treatment (three species of vegetables and two localities) and were expressed as the mean values and standard deviation (SD). All data were checked for homogeneity of variance and normality by Levene and Shapiro–Wilk tests. Data met assumptions for the use of one-way analysis of variance (ANOVA). ANOVA with Tukey’s test (*p* < 0.05) was used to identify statistically significant differences between the soils for each vegetable (lower-case letters) and among vegetable species for each soil (upper-case letters). The relationships between the content of toxic elements in the soil and determined parameters were assessed by principal component analysis (PCA) using the CANOCO 4.5 program [29].

## 3. Results

### 3.1. Toxic Elements in Vegetables

**Figure 1:**
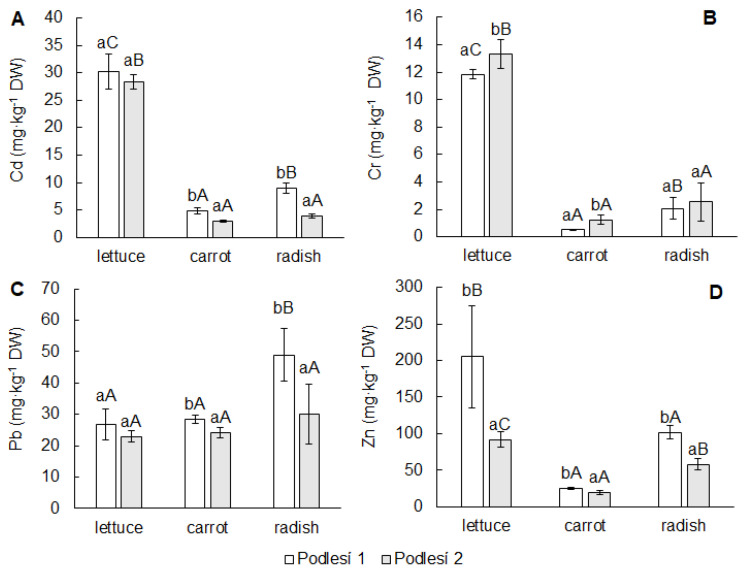
Contents of Cd (**A**), Cr (**B**), Pb (**C**) and Zn (**D**) in edible parts of lettuce, carrot and cherry radish. Values represent the mean ± SD (*n* = 4). Data with the same letter are not significantly different. Different letters indicate significant differences between the soils for each vegetable (lower-case letters) and among vegetable species for each soil (upper-case letters) according to one-way ANOVA with Tukey’s post hoc test (*p* < 0.05).

The contents of toxic elements in edible parts were dependent on element concentration in soil, their chemical forms, element mobility and the plant species. Soil from Podlesí 1 contained significantly more Cd, Pb and Zn compared to the soil from Podlesí 2. The contents of Cr were not different (Table 1). Differing soil contamination mainly affected the contents of toxic elements in root vegetables. The differences in the contents of toxic elements between leafy and root vegetables were confirmed (Figure 1A–D). The highest contents of Cd (28 and 30 mg·kg^−1^ DW), Zn (92 and 205 mg·kg^−1^ DW) and Cr (12 and 13 mg·kg^−1^ DW) were determined in the leaves of lettuce grown in both soils. Lead was especially accumulated in carrot roots (24 and 29 mg·kg^−1^ DW) and radish tubers (30 and 49 mg·kg^−1^ DW).

### 3.2. Bioaccumulation Factor

**Figure 2:**
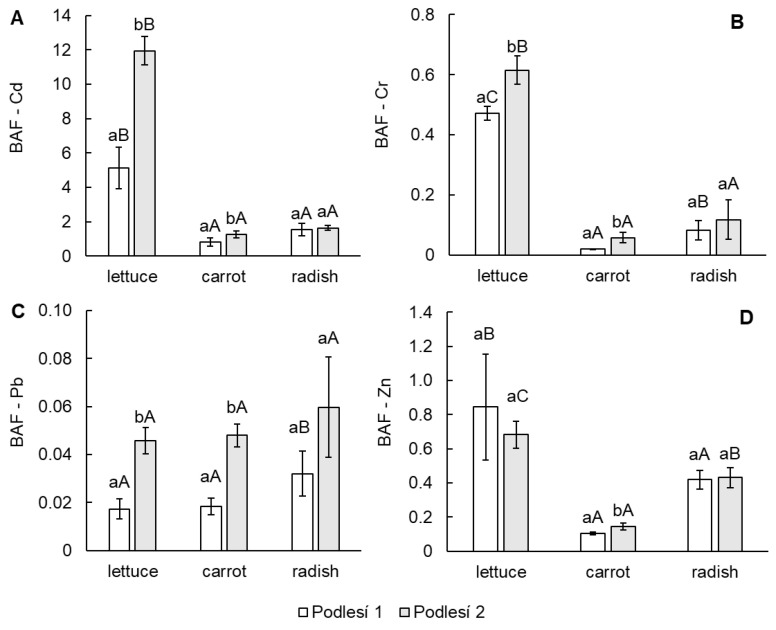
Bioaccumulation factor (BAF is the content of the element in edible part/content of the vegetable in soil) of Cd (**A**), Cr (**B**), Pb (**C**) and Zn (**D**) in the edible parts of lettuce, carrot and cherry radish. Values represent the mean ± SD (*n* = 4). Data with the same letter are not significantly different. Different letters indicate significant differences between the soils for each vegetable (lower-case letters) and among vegetable species for each soil (upper-case letters) according to the one-way ANOVA with Tukey’s post hoc test (*p* < 0.05).

From the data in Figure 2A–D, it can be observed that the BAF of vegetables increased in the order of Cd > Zn > Cr > Pb. The differences in BAF of Cd, Cr and Zn between leafy and root vegetables were confirmed. The bioaccumulation factor for Pb was affected by low element mobility in soil.

### 3.3. Chronic Daily Intake

**Figure 3:**
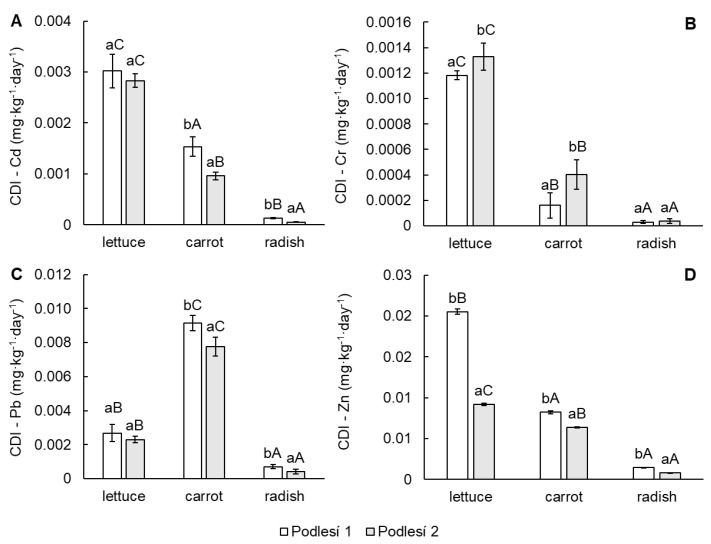
Chronic daily intake (CDI) of Cd (**A**), Cr (**B**), Pb (**C**) and Zn (**D**) in the edible parts of lettuce, carrot and cherry radish. Values represent the mean ± SD (*n* = 4). Data with the same letter are not significantly different. Different letters indicate significant differences between the soils for each vegetable (lower-case letters) and among vegetable species for each soil (upper-case letters) according to one-way ANOVA with Tukey’s post hoc test (*p* < 0.05).

The daily intake of toxic elements depended on the concentration of elements in the vegetable (Figure 1A–D) and the daily vegetable consumption. Data on the consumption of individual vegetables used for calculation were published by the Ministry of Agriculture of the Czech Republic [26]. The results of CDI for each element are shown in Figure 3A–D. The calculated values of CDI decreased in the order Zn > Pb > Cd > Cr. The higher sources of toxic elements in the human diet were lettuce leaves and carrot roots in contrast to radish tubers.

### 3.4. Risk Assessment

Risk to human health from the intake of toxic elements accumulated in the edible parts of vegetables was characterized using THQ. The target hazard quotient varied from 0.0054 to 3.019 for Cd, and it was 0.122 to 2.613 for Pb, 0.0028 to 0.0684 for Zn and 0.0099 to 0.443 for Cr. Among vegetables, the lowest values of THQ were calculated for all elements in radish tubers, and the highest values were for lettuce leaves. The exception is Pb, where the highest value of THQ (2.613) was determined in carrot roots. Among the four elements studied, Cd and Pb showed the highest THQ values for all vegetables.

**Figure 4:**
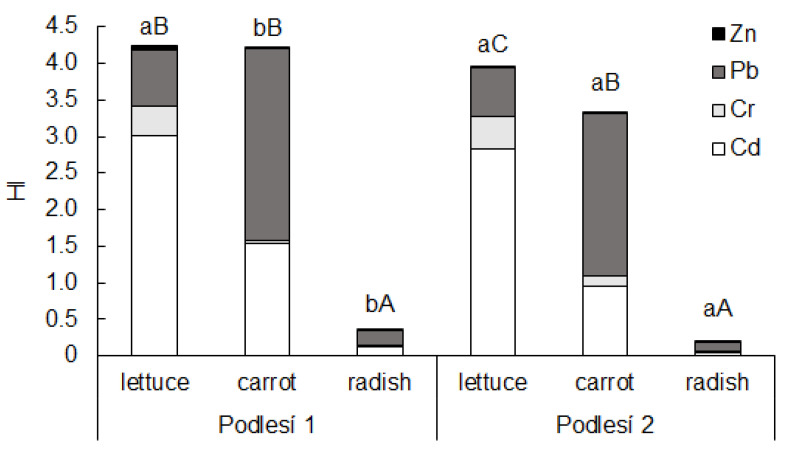
Hazard index (HI) of Cd, Cr, Pb and Zn in the edible parts of lettuce, carrot and cherry radish. Values represent the mean (*n* = 4). Data with the same letter are not significantly different. Different letters indicate significant differences between the soils for each vegetable (lower-case letters) and among vegetable species for each soil (upper-case letters) according to one-way ANOVA with Tukey’s post hoc test (*p* < 0.05).

The average THQ of the toxic elements decreased in the order of Cd > Pb > Cr > Zn. The total HI values higher than 1 were calculated for lettuce and carrot (Figure 4). The contribution of each evaluated element to the calculated HI revealed that Cd (contribution up to 75% in lettuce) and Pb (contribution up to 61% in carrot) were the most important elements posing a health risk. The cumulative effect of the consumption of these vegetables with HI > 1 showed possible non-carcinogenic health effects [28].

**Figure 5:**
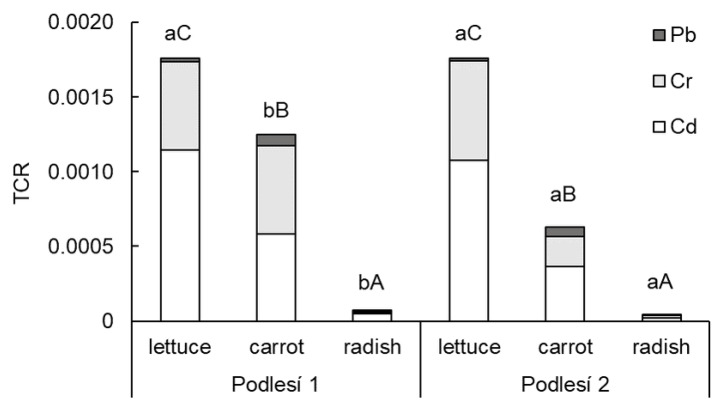
Total carcinogenic risk (TCR) of Cd, Cr, Pb and Zn in the edible parts of lettuce, carrot and cherry radish. Values represent the mean (*n* = 4). Data with the same letter are not significantly different. Different letters indicate significant differences between the soils for each vegetable (lower-case letters) and among vegetable species for each soil (upper-case letters) according to one-way ANOVA with Tukey’s post hoc test (*p* < 0.05).

The CR values of toxic elements decreased in the order of Cd > Cr > Pb. The higher CR values of Cd and Cr (0.00054 and 0.00026, respectively) were calculated and compared with a threshold value of 10^−4^ cancer risk [28]. The carcinogenic risk value of Pb was determined lower compared to Cd or Cr at 0.00003. The values of TCR (Figure 5) showed a carcinogenic risk through the consumption of lettuce and carrot. The TCR for radish was lower than 10^−4^.

### 3.5. Macronutrients

The content of macroelements in plants is dependent upon the amount of an element available in the soils and the relationship with other elements. The significant effect of soil contamination on the S content of vegetables was confirmed. A higher S content was determined in all vegetables grown in the Podlesí 1 locality. The content of this element was also different among vegetable species. Higher contents of Ca and Mg were determined in lettuce in contrast to both root vegetables (Table 3).

### 3.6. Free Amino Acids

**Figure 6:**
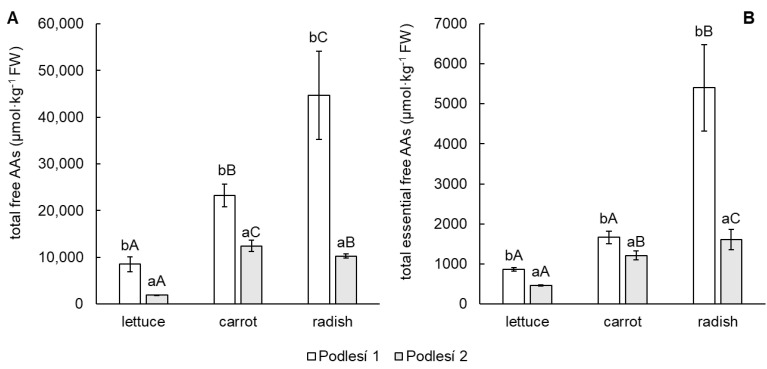
Total content of free amino acids (**A**) and essential free amino acids (**B**) in the edible parts of lettuce, carrot and cherry radish. Values represent the mean ± SD (*n* = 4). Data with the same letter are not significantly different. Different letters indicate significant differences between the soils for each vegetable (lower-case letters) and among vegetable species for each soil (upper-case letters) according to one-way ANOVA with Tukey’s post hoc test (*p* < 0.05).

The total content of free AAs was significantly higher in radish tubers in soil from the Podlesí 1 locality (5.2-fold and 1.9-fold higher compared to lettuce and carrot, respectively), while the content of free AAs from the Podlesí 2 soil was the highest in carrot (6.4-fold and 1.2-fold higher compared to lettuce and radish, respectively), as shown in Figure 6A. Contents of individual free AAs are presented in Table S2.

The amounts of essential free AAs (histidine, isoleucine, leucine, lysine, methionine, phenylalanine, threonine and valine) reached 10 and 24% of total free AAs in lettuce, 7 and 10% of total free AAs in carrot and 12 and 16% of total free AAs in radish in the Podlesí 1 and Podlesí 2 soils, respectively. In all edible parts of vegetables, the content of essential free AAs was significantly different between soils. For vegetables grown in both soils, the content of the essential free AAs group was highest in radish compared to lettuce and carrot (Figure 6B). The main free AAs of this group were threonine and valine in all vegetable species grown in soil from both soils. The content of threonine reached 22 to 35% of the essential AA total contents, and valine reached 21 to 37% of the essential AA total contents. The highest contents of these two AAs were determined in radish grown in both soils. Other essential free AAs with high abundance were isoleucine and histidine, especially in radish.

### 3.7. Relationship between Toxic Elements in Soil and Determined Parameters

**Figure 7:**
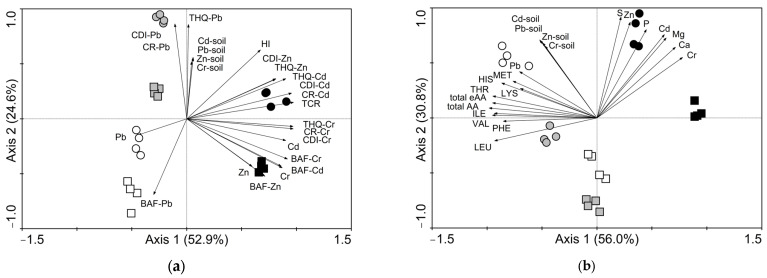
(**a**) Principal component analysis (PCA) for toxic element contents in the soil and determined health risk parameters in the edible parts of lettuce, carrot and cherry radish; (**b**) principal component analysis (PCA) for toxic element contents in the soil and determined parameters of quality assessment in the edible parts of lettuce, carrot and cherry radish. The length and direction of the vectors indicate the strength of the vector effect and the correlation between the vectors, respectively. A long vector for a particular variable indicates that it greatly affects the results of the analysis, while the opposite is true for a short vector. An angle of <90° between the vectors indicates that they are positively correlated. An angle of >90° between two vectors indicates that they are not positively correlated. Abbreviations: Cd, Cr, Pb, Zn, S, Ca, Mg, P—the contents of elements in the edible parts of vegetables; Cd-soil, Cr-soil, Pb-soil, Zn-soil—pseudo-total contents of toxic elements in the soil; BAF-Cd, BAF-Cr, BAF-Pb, BAF-Zn—bioaccumulation factors of toxic elements in the edible parts of vegetables; CDI-Cd, CDI-Cr, CDI-Pb, CDI-Zn—chronic daily intake values of toxic elements in the edible parts of vegetables; CR-Cd, CR-Cr, CR-Pb—carcinogenic risk levels of toxic elements in the edible parts of vegetables; TCR—total carcinogenic risk levels of the edible parts of vegetables; THQ-Cd, THQ-Cr, THQ-Pb, THQ-Zn—target hazard quotients of toxic elements in the edible parts of vegetables; HI—hazard index of the edible parts of vegetables; total eAA—total contents of essential free amino acids in the edible parts of vegetables; total AA—total contents of free amino acids in the edible parts of vegetables; HIS—free histidine in the edible parts of vegetables; MET—free methionine in the edible parts of vegetables; THR—free threonine in the edible parts of vegetables; ILE—free isoleucine in the edible parts of vegetables; VAL—free valine in the edible parts of vegetables; PHE—free phenylalanine in the edible parts of vegetables; LEU—free leucine in the edible parts of vegetables.

PCA analysis was used for the determination of relationships between the contents of toxic elements in the soil and the parameters of the risk assessment of edible parts of individual vegetables and soils (Figure 7a), as well as the quality assessment parameters of edible vegetable parts (Figure 7b).

The PCA analysis visualized in the PCA diagram (Figure 7a) showed that the first and second ordination axis explained 77.5% of all analyzed data variability. The symbols of the root vegetable group are located on the left side of diagram from the leafy vegetable group on the right side of diagram. This division indicates a large effect of vegetable type and toxic element contamination on the studied risk assessment parameters. A similar trend was observed in the case of quality assessment parameters, where the first and second ordination axis explained 86.8% of all analyzed data variability. Similarly, symbols are divided to opposite sides in the diagram of parameters of quality assessment—the root vegetable group on the left side and the leafy vegetable group on the right side.

## 4. Discussion

Vegetables, especially leafy vegetables, cultivated in contaminated soils absorb high amounts of toxic elements, with most of the absorbed elements accumulating in the leaves [12]. Lettuce belongs to leafy vegetables, which have the highest capacity of accumulating Cd from contaminated soil [7,9,10] and can contribute the greatest to human health risks [11]. Our results were consistent with these findings and confirmed the highest contents of Cd, Zn and Cr in leaves of lettuce. Lettuce accumulates high toxic element concentrations in its leaves due to both root uptake from contaminated soil and foliar uptake from soil dust and particulate matter [30].

Different contents of Zn in vegetables are given by growing conditions and varieties of individual vegetables. Zinc concentrations in food plants from various countries were found to fall within 44–73 mg·kg^−1^ for mean non-toxic values for lettuce and 21–27 mg·kg^−1^ for carrot roots [31,32]. Knez et al. [33] demonstrated similar Zn content in carrot root (2 mg·kg^−1^ in FW and in 11.7% DW). Toxic Zn concentrations in the leaves of plants range from 100 to 300 mg·kg^−1^ DW [34]. Similar values were determined for lettuce leaves in our experiment and reached 92–205 mg·kg^−1^ DW. Increased Zn contents in vegetables and fruits were also reported for metal-polluted areas from Romania [35].

The BAF is a parameter reflecting the ability of toxic elements to transfer from soil to plants. This value is affected by the ability of plants to assimilate toxic elements. The highest BAF value was calculated for Cd. The present study showed that the leafy vegetable lettuce had higher a Cd enrichment capacity than root vegetables. Some previous studies showed that leafy vegetables were more likely to accumulate Cd than non-leafy vegetables [36], which is consistent with the results of the present study. The BAF was also affected by the bioavailability of elements in soil and their interactions. The high value of BAF for Cd in our results was associated with a high portion of water-soluble Cd in contrast with other tested elements. Cadmium has high soil bioavailability compared to Pb and other toxic elements [37]. Interaction between Cd and Zn has a significant effect on Cd bioavailability for plants [38]. Lead was more accumulated in radish tubers and carrot roots in contrast to lettuce in our experiment. Bidar et al. [7] confirmed root vegetables as moderate Pb accumulators. According to other studies [5,6], Pb in most plants is accumulated in roots. Dogan et al. [39] published that Pb accumulates primarily in root cells because of the blockage by Casparian strips.

The ingestion of vegetables cultivated on soil contaminated with toxic elements causes serious human health issues, such as gastrointestinal cancer, fragile immunological mechanisms, mental growth retardation and malnutrition [20]. The calculated values of CDI and THQ for the four tested toxic elements suggest that the consumption of edible parts of vegetables by adults in the Příbram region is likely to lead to adverse health effects. The basic source of toxic elements in the human diet was lettuce and carrot. Also, Gupta et al. [19] found the highest CDI value for leafy vegetables, especially spinach. The low CDI value through tubers of radish is affected by the low consumption of this vegetable.

In our experiment, the risk to human health from the intake of toxic elements accumulated in edible parts of vegetables was characterized using THQ. Among vegetables, the highest values of THQ were calculated for all elements in lettuce. The exception is Pb—the highest value of THQ was determined in carrot roots. Zheng et al. [40] confirmed the highest risk of Cd ingestion from leafy vegetable consumption in parts of Huizhou, China. The health risks varied among crop species. The THQs were sorted by vegetable category in decreasing order: leafy vegetables > root vegetables > legume vegetables > cucurbit fruiting vegetables > stalk and stem vegetables. Our results are in accordance with this finding for Cd in relation to the risk of leafy vegetables. In contrast to our results, Zheng et al. [40] also indicated leafy vegetables as a high risk for Pb than other vegetables. Our results did not confirm this finding and showed carrot as the vegetable with the highest Pb risk. THQ values higher than 1 for carrot and spinach were confirmed by Antoniadis et al. [41] and Gupta et al. [19]. Among the four studied elements, Cd and Pb showed the highest THQ values for all vegetables. Both elements are carcinogenic for humans.

Total HI values higher than 1 were calculated for lettuce and carrot. Lead and Cd were found to be the elements with the most important contribution to total HI compared to the other toxic elements; this finding concurs with other authors [40,42,43]. These results clearly demonstrate that the adult population of the Příbram area is at high risk if lettuce and carrot are consumed as part of a daily diet [18,19]. The cumulative effect of consumption of these vegetables with HI > 1 can have an adverse non-carcinogenic health effect. Our results for lettuce and carrot showed HI values of 3.5–4.2, and according to Li et al. [44], a HI value of 1–5 indicates a concerning health hazard level. A value of HI > 10 indicates chronic health hazards. On the other hand, according to Zheng et al. [40], a value of HI > 1 may not indicate that a population suffered high health exposure risks from crop consumption because not all contents of toxic elements entering the human body through food consumption are bioavailable. Approximately 40% of the element contents are absorbed by the gastrointestinal tract.

The carcinogenic effect analysis revealed potential cancer risks posed by Cd, Cr and Pb from the consumption of lettuce and carrot. Their CR values were higher than the maximum threshold value of 10^−4^ [28]. A similar finding was published by Gebeyehu and Bayissa [45] for cabbage and tomato cultivated in Ethiopia and the elements As, Cd and Cr. Our results suggest the presence of potential cancer risk to the adult population from tested elements due to the consumption of both vegetables cultivated in the Příbram region. Based on our results, we can confirm there is a significant health risk (both non-carcinogenic and carcinogenic) to humans associated with the consumption of lettuce and carrot cultivated on contaminated soils in the Příbram region.

Based on vegetables being important components of the human diet, it is important to assess their quality, especially in contaminated soil. Vegetables are important sources of minerals in the human diet. The content of minerals in plants is dependent upon the amount of an element available in the soil for the growing plant. Plants that were exposed to toxic elements have shown changes in the content of macro- and microelements in biomass [24,46]. Our results did not confirm different contents of macroelements in the edible parts of root vegetables grown in soils with different contamination levels and showed results for Ca and Mg similar to Knez et al. [33]. The Ca content in the leaves of lettuce ranges from 4 to 16 mg·g^−1^ DW [47]. Lettuce is generally low in P and Mg compared to other leafy vegetables, such as spinach, which has 0.9–2.5 and 2.1–9.5 mg·g^−1^ DW of P and Mg, respectively [47]. Sulfur is associated with plant tolerance against abiotic stress. An important way to avoid stress is the chelation of toxic elements by high-affinity ligands. Ligands include amino acids, organic acids and two groups closely related to S: metallothioneins and their class III—phytochelatins [48]. Colovic et al. [49] documented the importance of S for plant resistance against the possible influence of heavy metals. Shi et al. [50] published that sufficient S supply in solutions contaminated by high Cd doses significantly alleviated Cd toxicity for wheat seedlings and its translocation by increasing phytochelatin synthesis and the subsequent vacuolar sequestration of Cd in roots. The significant effect of soil contamination on S content in vegetables was confirmed, and the contents of this element were also different among vegetable types in our experiment.

The content of AAs in the plant can be affected by the environment. AAs are crucial for the human diet because AAs used in protein synthesis cannot be synthesized in the human body. Vegetables are mostly low in specific essential AAs [51]. On the other hand, plants exposed to toxic elements have been shown to accumulate specific AAs, which may play an important role in their detoxification [17,52]. The accumulation of free AAs in plants can be the result of a decrease in their degradation, an increase in AA synthesis or the hydrolysis of proteins during plant stress responses [53]. According to Chaffei et al. [54], an increase in the proportion of high N:C amino acids is a protective strategy in plants for preserving roots. The results of Zemanová et al. [55] indicated the accumulation of large amounts of AAs in the roots of plants stressed by toxic elements in pot experiments. Our results are consistent with these findings. The higher contents of total free AAs were determined in all vegetables grown in Podlesí 1, which was a more contaminated locality, and the highest content of AAs was determined in radish tubers. Threonine, which has a significant role in plant responses to abiotic stress, causing energy deprivation [56], and valine, which is a building block of proteins in plants, were among the essential AAs increased in vegetables grown in contaminated soils. Pavlík et al. [57] published a dramatically increased content of valine in leaves of lettuce contaminated by dust. The higher content of histidine in the roots of *Noccaea caerulescens* in response to Cd stress indicates that this AA may be involved in Cd resistance and accumulation by reducing oxidative damage [55]. Similar data were found for radish tubers in our experiment.

## 5. Conclusions

This study provided insight into the scenario of vegetable contamination and human health risk estimations of contaminated areas. It revealed health risks and the quality reduction in vegetables cultivated in soils heavily polluted as a result of the atmospheric deposition of toxic elements (Cd, Cr, Pb and Zn) originating from historic lead–silver mining and smelting activities. Vegetable species, such as radish, carrot, and lettuce, were cultivated in a pot experiment using two soils from the Příbram region, one of the most polluted areas by toxic elements in the Czech Republic. To evaluate health risk assessment, chronic daily intake of toxic elements, the potential hazard to human health through the consumption of vegetables and carcinogenic risk were calculated. The quality reduction in vegetables was evaluated according to toxic element and macronutrient contents and changes in free amino acid content in their edible parts. We produced the following key conclusions: (1) The highest contents of Cd, Zn and Cr were determined in lettuce, and Pb was more accumulated in roots vegetables. (2) Cd was ranked as the highest risk among the toxic elements in cultivated vegetables. (3) The health risks varied among vegetables. The THQs were sorted in decreasing order: lettuce > carrot > radish. The low values for radish were influenced by the low consumption of this vegetable. These results clearly demonstrate that the adult population of this area is at a high risk (both non-carcinogenic and carcinogenic) if lettuce and carrot cultivated in the studied area are consumed as part of a daily diet. We can confirm there is a significant health risk; therefore, local vegetables are not suitable for long-term consumption and must be strictly controlled.

## Figures and Tables

**Table 1:** Basic characteristics and toxic element contents of experimental soils.

Parameters	Podlesí 1	Podlesí 2
Soil Type and Subtype	Cambisol Haplic	Cambisol Haplic
pH_H2O_	5.9	6.0
Cation Exchange Capacity (mmol_(+)_·kg^−1^)	165.8 ± 15.1	166.7 ± 12.3
Organic Carbon (%)	4.1 ± 0.1	4.8 ± 0.3
Pseudo-Total Content of Cd (mg·kg^−1^) ^1^	6.5 ± 1.1	2.4 ± 0.2
Pseudo-Total Content of Pb (mg·kg^−1^)	1560.2 ± 167.1	503.6 ± 14.3
Pseudo-Total Content of Cr (mg·kg^−1^)	24.9 ± 1.0	22.4 ± 1.6
Pseudo-Total Content of Zn (mg·kg^−1^)	243.4 ± 8.2	135.0 ± 0.6
Water-soluble Content of Cd (mg·kg^−1^)	0.01 ± 0.001	0.007 ± 0.001
Water-soluble Content of Pb (mg·kg^−1^)	0.4 ± 0.04	0.3 ± 0.03
Water-soluble Content of Cr (mg·kg^−1^)	0.03 ± 0.001	0.03 ± 0.001
Water-soluble Content of Zn (mg·kg^−1^)	0.2 ± 0.03	0.1 ± 0.01

Czech legislation limits for pseudo-total contents of elements in light-textured and other soils are 0.4 and 0.5 mg·kg^−1^ for Cd, 55 and 60 mg·kg^−1^ for Pb, 55 and 90 mg·kg^−1^ for Cr, 105 and 120 mg·kg^−1^ for Zn, respectively [23].

**Table 2:** The parameters used in the calculation of bioaccumulation factors and health risk assessment.

BAF	Bioaccumulation Factor	
C_vge_	Content of Toxic Elements in The Edible Parts of Vegetables	mg·kg^−1^ dry weight (DW)
C_soil_	Content of Toxic Elements in Soils	mg·kg^−1^ soil
CDI	Chronic Daily Intake of Toxic Elements	
IR	Daily Consumption of Vegetables	kg·person^−1^·day^−1^
EF	The Exposure Frequency	365 days
ED	Exposure Duration	30 years for adult
ET	The Exposure Time ED × 365	days
BW	Body Weight	70 kg for adult
THQ	Target Hazard Quotient	
RfD	The Oral Reference Consumption Dose of Toxic Elements	mg·kg^−1^·day^−1^
HI	Hazard Index	
CR	Carcinogenic Risk	
SF	The Slope Factor of Carcinogenic Elements	mg·kg^−1^·day^−1^
TCR	Total Carcinogenic Risk	

**Table 3:** Contents of macronutrients in the edible parts of lettuce, carrot and cherry radish.

Vegetable	Soil	Ca (% DW)	Mg (% DW)	P (% DW)	S (% DW)
Lettuce	Podlesí 1	2.16 ± 1.32 ^aB^	0.52 ± 0.09 ^bB^	0.26 ± 0.03 ^aB^	0.20 ± 0.02 ^bC^
	Podlesí 2	1.57 ± 0.22 ^aB^	0.34 ± 0.05 ^aB^	0.26 ± 0.02 ^aB^	0.14 ± 0.01 ^aC^
Carrot	Podlesí 1	0.22 ± 0.02 ^aA^	0.10 ± 0.01 ^aA^	0.15 ± 0.01 ^aA^	0.06 ± 0.003 ^bA^
	Podlesí 2	0.24 ± 0.01 ^aA^	0.11 ± 0.01 ^aA^	0.15 ± 0.01 ^aA^	0.04 ± 0.003 ^aA^
Radish	Podlesí 1	0.42 ± 0.03 ^aA^	0.18 ± 0.01 ^aA^	0.22 ± 0.02 ^bB^	0.16 ± 0.01 ^bB^
	Podlesí 2	0.43 ± 0.03 ^aA^	0.15 ± 0.03 ^aA^	0.14 ± 0.01 ^aA^	0.09 ± 0.02 ^aB^

Notes: values represent the mean ± SD (*n* = 4). Data with the same letter are not significantly different. Different letters indicate significant differences between the soils for each vegetable (lower-case letters) and among vegetable species for each soil (upper-case letters) according to one-way ANOVA with Tukey’s post hoc test (*p* < 0.05).

## Data Availability

The data presented in this study are available in the article.

## Supplementary Materials

The following supporting information can be downloaded at: https://www.mdpi.com/article/10.3390/toxics11070583/s1, Figure S1: Location of the studied area; Figure S2: Yield of edible parts of vegetable; Table S1: Accuracy assessment using the analysis of spinach and tomato leaves; Table S2: Contents (μmol·kg^−1^ FW) of free amino acids and related compounds in edible parts of vegetables.
