# Supplementary material for: Health Risk and Quality Assessment of Vegetables Cultivated on Soils from a Heavily Polluted Old Mining Area
Source: Toxics. 2023 Jul 4;11(7):583. doi: 10.3390/toxics11070583 (PMC10384379; doi:10.3390/toxics11070583)
Supplement: Supplementary file 1 [file toxics-11-00583-s001.zip › toxics-2398941-supplementary.pdf]

Table S1. Accuracy assessment using the analyses of spinach and tomato leaves (CRM NIST 1570a spinach leaves; CRM NIST 1573a tomato leaves).

Element	Certified value	Information value	Found value
Cd (mg/kg DW)[a]	2.88 ± 0.06	-	2.71 ± 0.21
Cr (mg/kg DW)[b]	4.5 ± 0.5	-	3.50 ± 0.29
Pb (mg/kg DW)[b]	6.3 ± 0.3	-	5.86 ± 0.31
Zn (mg/kg DW)[a]	82.3 ± 3.9	-	79.53 ± 2.69
Ca (% DW)[a]	1.53 ± 0.07	-	1.46 ± 0.02
Mg (% DW)[a]	-	0.9	0.87 ± 0.01
P (% DW)[a]	0.52 ± 0.01	-	0.51 ± 0.03
S (%DW)[a]	-	0.5	0.42 ± 0.04

[a] CRM NIST 1570a spinach leaves; [b] CRM NIST 1573a tomato leaves.

Table S2. Contents (μmol·kg^{-1} FW) of free amino acids and related compounds in edible parts of vegetables.

	Lettuce		Carrot		Radish	
	Podlesí 1	Podlesí 2	Podlesí 1	Podlesí 2	Podlesí 1	Podlesí 2
α-aminoadipic acid	142.7 ± 21.5	30.0 ± 0.9	52.4 ± 6.2	54.0 ± 2.5	93.1 ± 11.5	58.2 ± 3.5
γ-aminobutyric acid	47.5 ± 8.3	11.9 ± 0.5	30.0 ± 2.4	28.0 ± 1.4	22.4 ± 2.5	23.2 ± 1.7
Alanine	351.1 ± 38.5	37.4 ± 2.1	768.1 ± 309.4	525.0 ± 88.0	882.7 ± 193.5	237.6 ± 17.4
Asparagine (amide)	2469.9 ± 438.3	45.9 ± 7.4	3347.1 ± 141.0	1978.8 ± 210.6	1419.7 ± 303.2	267.6 ± 37.8
Aspartic acid	376.8 ± 70.0	41.1 ± 2.0	2911.5 ± 364.4	1576.8 ± 139.6	4311.6 ± 982.8	1254.1 ± 85.0
Glutamine (amide)	4811.8 ± 1329.1	114.2 ± 9.6	11481.6 ± 915.4	5332.4 ± 408.3	17273.9 ± 3754.8	3462.8 ± 651.7
Glutamic acid	642.0 ± 79.6	139.7 ± 8.4	1565.1 ± 149.5	1087.9 ± 96.6	3494.5 ± 464.4	1455.6 ± 185.4
Glycine	117.2 ± 5.0	14.4 ± 0.6	83.9 ± 6.6	67.5 ± 8.2	295.8 ± 43.2	111.9 ± 16.3
Glycine–proline (dipeptide)	nd	nd	71.5 ± 9.2	nd	61.2 ± 1.1	79.6 ± 29.8
histidine	272.9 ± 18.6	18.4 ± 0.9	105.8 ± 2.5	61.1 ± 5.6	669.6 ± 130.9	133.9 ± 12.5
4-hydroxyproline	nd	nd	nd	nd	52.1 ± 9.2	43.5 ± 4.3
Isoleucine	291.0 ± 16.0	34.8 ± 6.4	183.5 ± 15.5	142.1 ± 4.0	860.4 ± 159.2	196.5 ± 24.8
Leucine	208.5 ± 24.2	30.7 ± 2.7	113.7 ± 9.6	103.2 ± 7.2	175.1 ± 26.0	95.3 ± 9.2
Lysine	217.5 ± 13.8	43.8 ± 2.2	90.5 ± 5.3	100.1 ± 6.0	337.7 ± 85.7	111.6 ± 5.0
Methionine	138.0 ± 24.1	20.2 ± 0.9	77.7 ± 3.6	51.6 ± 3.0	70.6 ± 7.8	45.0 ± 3.0
Ornithine	158.1 ± 27.0	42.9 ± 2.1	62.8 ± 7.9	81.5 ± 3.3	73.0 ± 10.1	78.9 ± 0.9
Phenylalanine	113.0 ± 15.9	11.2 ± 2.5	95.4 ± 5.9	80.1 ± 9.2	84.7 ± 15.7	39.9 ± 4.7
Proline	516.7 ± 175.1	14.7 ± 0.6	449.8 ± 235.6	80.5 ± 1.2	9554.2 ± 1600.4	822.5 ± 391.1
Sarcosine	157.0 ± 28.9	12.3 ± 0.6	nd	nd	nd	nd
Serine	768.7 ± 107.4	103.5 ± 4.2	571.8 ± 113.4	292.4 ± 46.6	1501.8 ± 457.6	542.6 ± 75.8
Threonine	637.9 ± 105.2	59.1 ± 5.5	485.1 ± 52.6	291.9 ± 33.1	1218.3 ± 205.5	452.8 ± 77.8
Tryptophan	228.8 ± 34.5	31.7 ± 5.4	94.6 ± 5.6	39.8 ± 8.3	116.8 ± 19.5	117.5 ± 14.5

Tyrosine	181.1 ± 28.1	26.9 ±0.6	99.1 ± 3.6	74.1 ± 8.3	124.9 ± 23.6	89.1 ± 7.5
Valine	775.1 ± 78.2	91.3 ± 9.3	510.8 ± 58.1	380.4 ± 32.6	1984.8 ± 341.0	535.5 ± 89.3

nd—not detected.

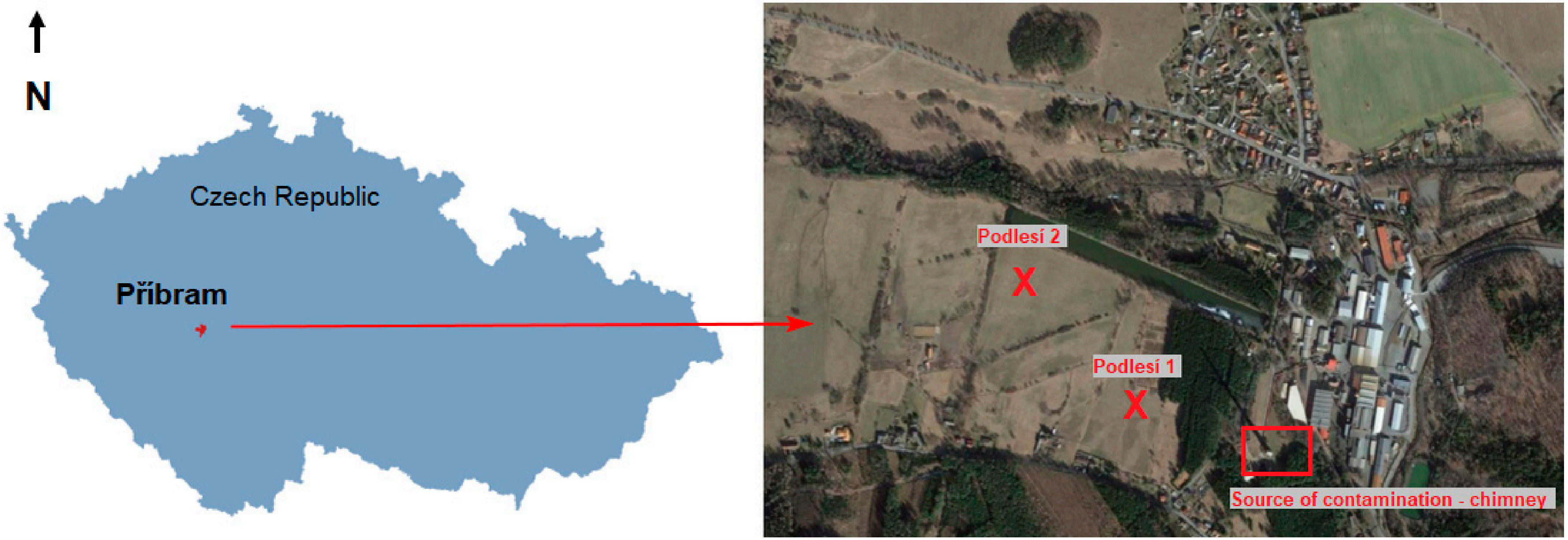

Figure S1. Location of the studied area.

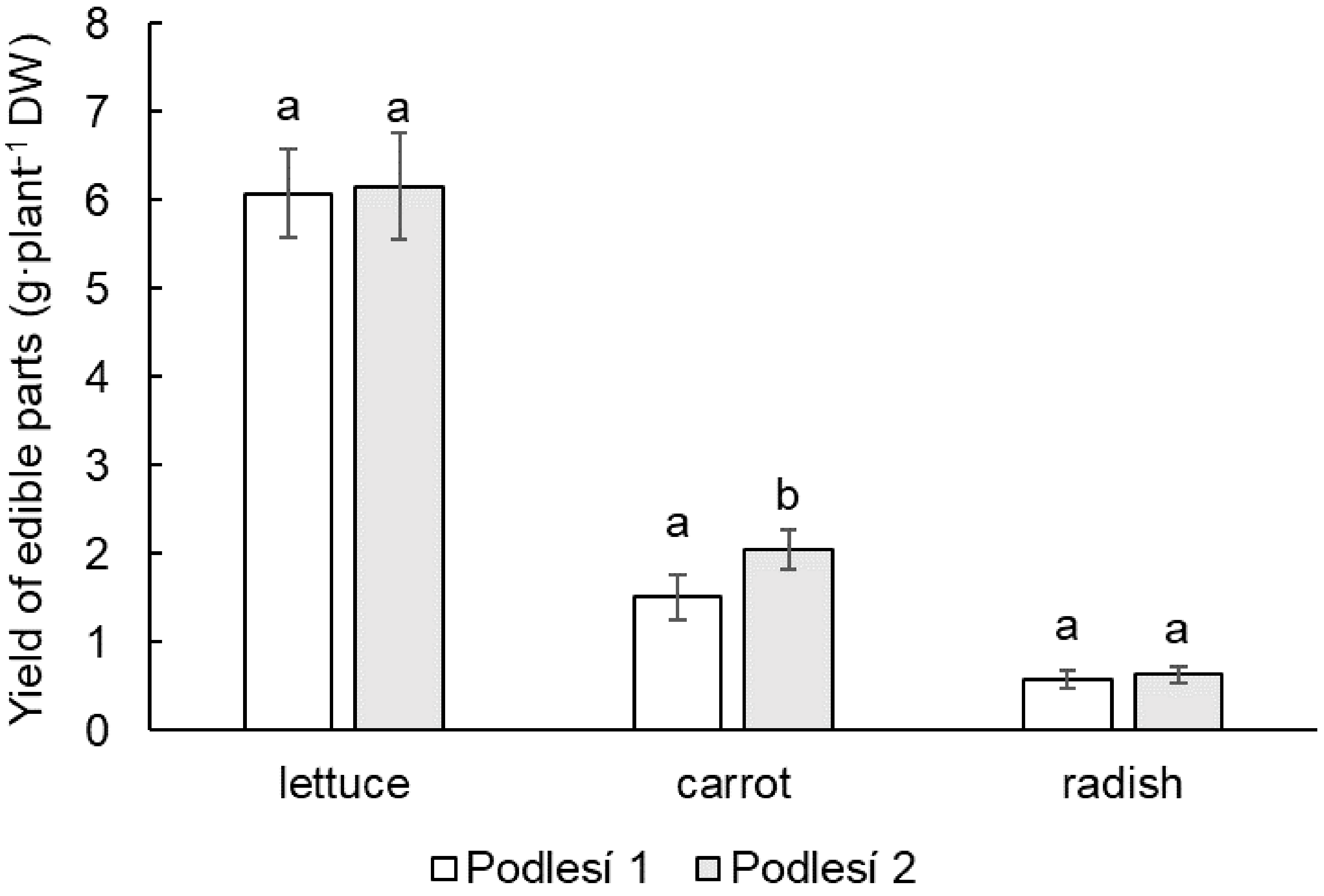

Figure S2: Yield of edible parts of vegetable.
